# Artificial intelligent patient-controlled intravenous analgesia improves the outcomes of older patients with laparoscopic radical resection for colorectal cancer

**DOI:** 10.1007/s41999-023-00873-z

**Published:** 2023-10-17

**Authors:** Dandan Liu, Xiaopei Li, Xiaohong Nie, Qiangfu Hu, Jiandong Wang, Longzhu Hai, Lingwei Yang, Lin Wang, Peilei Guo

**Affiliations:** 1 Department of Surgery, The Fifth Affiliated Hospital of Zhengzhou University, Zhengzhou, Henan China; 2 Department of Anesthesiology, The Fifth Affiliated Hospital of Zhengzhou University, No. 3, Kangfuqian Street, Erqi District, Zhengzhou, Henan China

**Keywords:** Patient-controlled analgesia, Surgery, Colorectal cancer, Care

## Abstract

**Methods:**

Patients undergoing elective laparoscopic radical resection of colorectal cancer from July 2019 to May 2021 were selected. The patients were assigned to Ai-PCIA group and control group. Ai-PCIA group received postoperative analgesia management and effect evaluation through intelligent wireless analgesia system + postoperative follow-up twice a day, while control group received analgesia management and effect evaluation through ward physician feedback + postoperative follow-up twice a day. The pain numerical score (NRS), Richards–Campbell Sleep Scale (RCSQ), and adverse outcomes were collected and compared.

**Results:**

A total of 60 patients (20 females and 40 males with average (78.26 ± 6.42) years old) were included. The NRS scores at rest and during activity of the Ai-PCA group at 8, 12, and 24 h after the operation were significantly lower than that of the control group (all *P* < 0.05). The RCSQ score of Ai-PCA group was significantly higher than that of control group on the 1st and 2nd days after operation (all *P* < 0.05). There were no significant differences in the incidence of dizziness and nausea, vomiting, and myocardial ischemia (all *P* > 0.05).

**Conclusions:**

Ai-PCIA can improve the analgesic effect and sleep quality of older patients after laparoscopic radical resection, which may be promoted in clinical analgesia practice.

## Key summary points

**Aim:**

Artificial intelligent patient-controlled intravenous analgesia (Ai-PCIA) has been used in clinical anesthesia practice in recent years. We aim to evaluate the effect of Ai-PCIA in older patients after laparoscopic radical resection of colorectal cancer.

**Findings:**

Ai-PCIA improves the analgesic effect of older patients after laparoscopic radical resection of colorectal cancer, improve sleep quality, and improve the early postoperative outcome of older patients.

**Message:**

Ai-PCIA may be approperiate and promoted for clinical posoperative care in older patients after laparoscopic radical resection of colorectal cancer.

## Introduction

Surgery is the preferred treatment for early-stage colorectal cancer. With the development of minimally invasive surgical techniques, laparoscopic radical resection of colorectal cancer is widely used in clinical practice due to its characteristics of small trauma, low infection rate and quick postoperative recovery, but postoperative pain is still the main problem faced by patients [1–3]. Poor postoperative pain control can lead to complications such as depression, delirium, sleep disturbance, intestinal obstruction, and lung injury, affecting the recovery of body functions and prolonging hospitalization [4–6]. Therefore, the management and nursing care of analgesia after laparoscopic colorectal cancer surgery are of great significance to the prognosis of patients. However, there is still a lack of consensus on the optimal regimen for postoperative analgesia after laparoscopic colorectal cancer surgery, especially for the older patient.

It has been reported that early postoperative pain after laparoscopic radical resection of colorectal cancer is comparable to, or even more severe than, open surgery [7]. Poor postoperative pain control can increase postoperative complications, and may even turn into chronic pain and seriously affect prognosis [8]. Epidural analgesia has the risk of epidural hematoma and hypotension, and is not conducive to the early ambulation of patients and affects the recovery of body function [9–11]. Therefore, epidural analgesia is not recommended for pain management after laparoscopic colorectal surgery [12, 13]. At present, patient-controlled analgesia (PCA) has been widely used in the clinical analgesia. Although the traditional PCA management model can effectively achieve individualized analgesia, the analgesic effect may be affected by the inability to obtain key information in the analgesic management process in time [14–16]. Artificial intelligent PCA(Ai-PCA) is a new type of analgesia management system that combines traditional PCA with the Internet of Things and artificial intelligence [17, 18]. Several previous studies [19–22] have reported the effects of Ai-PCA in clinical analgesia, yet the results remain inconsistent. Currently, there are few studies on the effects of Ai-PCA in older patients undergoing laparoscopic radical resection of colorectal cancer. Therefore, we aimed to explore the effect of Ai-PCIA on postoperative analgesia in older patients undergoing laparoscopic radical resection of colorectal cancer, to provide useful implications for the clinical analgesia. This study hypothesized that Ai-PCIA may be more beneficial to improve the outcomes of older patients undergoing laparoscopic radical resection of colorectal cancer.

## Methods

### Ethic consideration

This study was reviewed and approved by the ethics committee of our hospital (approval number: KY2021040). In addition, we had informed the patient in detail about the possible risks and benefits of AI-PCIA intervention; all the included patients signed the informed consent.

### Sample size calculation

The formula of paired group sample size [23] was used for sample size calculation: $n = \frac{{\left( {u_{\alpha } + u_{\beta } } \right)^{2} 2p\left( {1 - p} \right)}}{{\left( {p_{1} - p_{2} } \right)^{2} }}$ . *p*_1_ was the incidence of unmet pain needs in the control group, *p*_2_ was the incidence of unmet pain needs in the intervention group, *p* = (*p*_1_ + *p*_2_)/2. *Z*_ɑ_ and *Z*_β_ were the corresponding values of the test level *α* and the type II error probability *β*. We assumed that *α* = 0.05, *β* = 0.10, two-sided test *Zα*/2 = 1.95. According to the results of the pre-experiment, *p*_1_ and *p*_2_ were 0.56 and 0.82, respectively, the calculated sample size of a single group was 26. Therefore, at least a total of 52 patients must be included for analysis.

### Patients

This study selected older patients who underwent elective laparoscopic radical resection of colorectal cancer in our hospital from July 2019 to May 2021 as the research population. The inclusion criteria for patients were: age ≥ 65 years old; patients with clear consciousness (Glasgow Coma Score = 15) and normal communication, and patients fully understood the numerical rating scale (NRS); patients were informed and agreed to participate in this study. The exclusion criteria of patients were: patients who used analgesics in recent one month, patients with a history of opioid abuse, patients with severe liver and kidney and other vital organ dysfunctions before surgery, and patients who were allergic to analgesic drugs; patients refused to use postoperative intravenous analgesia pump.

### Anesthesia method

After the patient entered the operating room, electrocardiogram (ECG) monitoring and temperature monitoring were performed, peripheral venous access was opened, invasive radial artery puncture under local anesthesia was performed to measure mean arterial pressure, right internal jugular vein puncture was performed, and central venous pressure was monitored. Anesthesia induction: intravenous midazolam 0.05 mg/kg, sufentanil 0.4–0.5 μg/kg, etomidate 0.15–0.30 mg/kg, vecuronium 0.08–0.10 mg/kg, and dexamethasone 5–10 mg. Mechanical ventilation was performed after tracheal intubation, and the oxygen flow was set at 2 L/min, tidal volume (VT) 8–10 ml/kg, respiratory rate (RR) 10–14 times/min, and partial pressure of end tidal carbon dioxide (P_ET_CO_2_) was maintained at 35–45 mmHg. Anesthesia maintenance: intravenous infusion of propofol 4–12 mg/kg/h, remifentanil 0.1–0.2 μg/kg/min and inhalation 0.6–1.5% sevoflurane to maintain bispectral index (BIS) 45–60. During the operation, vecuronium bromide was added intermittently to maintain muscle relaxation. The muscle relaxant was stopped 30 min before the end of the operation. All anesthetics were stopped after the operation, and the patient was sent to the post-anesthesia care unit (PACU).

### Patient assignment and management

Before surgery, all patients were given analgesia-related knowledge education, so that patients and their families could fully understand and master the NRS scoring method. The patients were assigned to the Ai-PCIA group and control group by random number table method. Ai-PCIA assigned to the intervention of intelligent wireless analgesia system + postoperative follow-up twice a day. Control group assigned to the intervention of analgesic pump, the dose and drugs of two group remained the same. The analgesia management and effect evaluation were carried out by means of ward physician feedback if the patient self-reported a high level of pain. Anesthesiologist performed postoperative follow-up at least twice a day. During PACU, after the patient's Steward score reaches 4 or more, the tracheal tube was removed and the analgesic pump was connected. All patients received the same analgesic pump formula, the analgesic pump formula was as follows: sufentanil 100 μg + dezocine 15 mg + azasetron 10 mg + normal saline to 100 ml, no loading dose, the background dose was 1.5 ml/h, the single additional dose was 1.5 ml, the locking time was 10 min, and the limit dose was 8 ml/h. The analgesic effect was evaluated after 15 min. If the NRS was ≥ 4 points, rescue analgesia was performed, that is, sufentanil 0.05 μg/kg was manually pumped through PCIA, and re-evaluated after 15 min, and the administration could be repeated. The vital signs were closely monitored after the drug use, and the patient returned to the ward when the NRS was less than 4 points.

The wireless analgesia system consisted of an analgesic pump, a base station, and a central information processing device (computer or mobile phone), which fed back the information of the running analgesic pump to the central information processing device through radio transmission for analysis, processing and generation of different alarm signals and sent it to the computer or mobile phone, and the anesthesiologist dealt with it in time according to the alarm signals (insufficient analgesia, poor analgesia, and blockage): (1) Insufficient analgesia: when the PCIA automatic control key was pressed 3 times ineffectively within the locking time, we timely gave rescue analgesia (the anesthesiologist manually pumped sufentanil 0.05 μg/kg through PCIA, and re-evaluated the analgesic effect after 15 min of observation; if the NRS score after rescue analgesia was ≤ 3 points, a single additional dose was administered. An increase of 0.1 ml was sufficient, the maximum was not more than 2 ml, and other parameters remained unchanged; if the NRS score was less than the score before rescue analgesia but still ≥ 4 points, intramuscular injection of dezocine 5 mg was given, and the parameters of the analgesic pump were adjusted. (2) Poor analgesia: the patients were educated that when the automatic control key was pressed for the fourth time within 1 h, the parameters of the analgesic pump would be adjusted in time (increased the single additional volume by 0.1 ml, the maximum should not exceed 2 ml, other parameters remained unchanged); (3) Blockage: when the external infusion pipeline of the analgesic pump was blocked, we investigated the reasons and deal with it in time. The routine follow-up was performed by the anesthesiologist between 8: 00–9: 00 and 17: 00–18: 00 in the two groups after operation. If the NRS score was ≥ 4, rescue analgesia was performed. During routine follow-up, if analgesic rescue had been performed according to the alarm signal or the ward physician's feedback, no additional administration was required to avoid repeated drug administration.

### Observation indicators

All the outcomes were evaluated and collected by three anesthesiologists, and we did not set blind design on the outcome assessment. We recorded and collected the intraoperative remifentanil dose and duration of surgery. NRS scores at rest and during activity (coughing or turning over) were recorded at 2, 4, 8, 12, 24, and 48 h after surgery. The total number of analgesic pump compressions, the total dose of sufentanil, the number of rescue analgesia cases, and the postoperative hospital stay of the patients within 48 h after operation were recorded. The Chinese version of the Richards Campbell sleep questionnaire (RCSQ) was used to evaluate the sleep status on the 1st day before surgery, the 1st day and the 2nd day after surgery. The RCSQ including sleep depth, difficulty falling asleep, number of awakenings, difficulty falling asleep again, and overall sleep quality, the RCSQ score is the average score of 5 items, of which 0–25 points represent poor sleep quality, and 76–100 points represent good sleep quality. Higher scores indicate better sleep quality. The RCSQ scores were collected at 8:00 a.m. for the last night sleep quality in both groups. The incidence of adverse reactions (nausea and vomiting, dizziness, respiratory depression, skin pruritus) and cardiovascular adverse events (myocardial ischemia, arrhythmia, unstable angina, acute myocardial infarction, and heart failure) within 48 h after surgery were recorded.

### Statistical analysis

This study used SPSS 26.0 software for data analysis. Normally distributed measurement data were expressed as mean ± standard deviation, and the comparison between groups was performed by two independent samples *t* test; non-normally distributed measurement data were expressed as the median (*M*) and interquartile range (IQR), and the comparison between groups was performed by Mann–Whitney *U* test. Enumeration data were expressed as cases (%), and comparisons between groups were performed using the Chi-square test or Fisher’s exact test. In this study, *P* < 0.05 indicated that the difference between groups was statistically significant.

## Results

### The characteristics of included patients

**Fig. 1:**
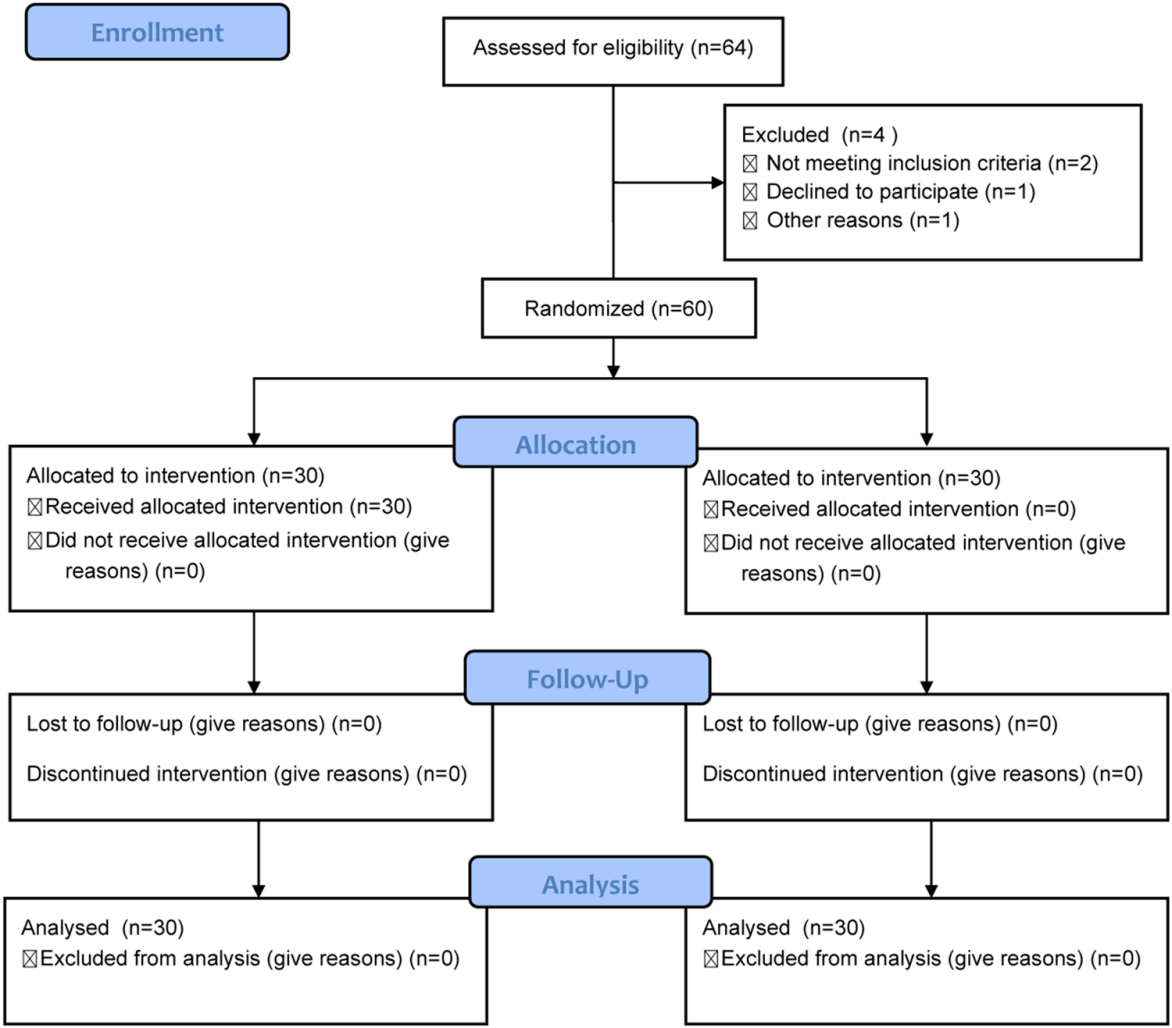
Flow diagram of patient selection

A total of 60 patients were included in this study (Fig. 1). As shown in Table 1, there were no significant differences in the gender, age, body mass index (BMI), ASA score, hypertension, diabetes, coronary heart disease, dosage of remifentanil, and duration of surgery between Ai-PCA group and control group(all *P* > 0.05).

**Table 1:** The characteristics of included patients

Characteristic	Ai-PCA group (*n* = 30)	Control group (*n* = 30)	*P*
Male/female	21/9	19/11	0.085
Age (y)	79.4 ± 6.6	76.1 ± 6.4	0.104
BMI (kg/m^2^)	23.7 ± 2.4	22.7 ± 3.0	0.092
ASA score			0.945
Level II	7 (23.3%)	7 (23.3%)	
Level III	23 (76.7%)	23 (76.7%)	
Hypertension	15 (50.00%)	14 (46.7%)	0.179
Diabetes	7 (23.3%)	9 (30.0%)	0.161
Coronary heart disease	5 (16.7%)	8 (26.7%)	0.097
Dosage of remifentanil (μg)	1900 (1500–2425)	1850 (1500–2500)	0.131
Duration of surgery (min)	230 (180–293)	220 (180–300)	0.083

*Ai-PCIA* artificial intelligent patient-controlled intravenous analgesia; *BMI* body mass index; *ASA* American Society of Anesthesiologists

### NRS scores

The resting NRS scores of the Ai-PCA group at 8, 12, and 24 h after the operation were significantly lower than that of the control group (more than 28.11%, all *P* < 0.05). At 8, 12, 24, and 48 h after operation, the NRS scores during activity of the Ai-PCA group were significantly lower than that of control group (more than 29.06%, all *P* < 0.05) (Table 2). The pain scores were generally quite low in this study.

**Table 2:** Comparison of NRS scores between the two groups of patients at rest and during activity at different time points after surgery [M(IQR)]

Status	Groups	2 h	4 h	8 h	12 h	24 h	48 h
At rest	Ai-PCA group (*n* = 30)	2 (1–2)	3 (2–4)	3 (2–4)*	4 (3–4)*	2 (2–3)*	1 (1–2)
	Control group (*n* = 30)	2 (0–2)	3 (2–4)	4 (3–5)	4 (4–5)	3 (2–4)	1 (0–2)
During activity	Ai-PCA group (*n* = 30)	3 (2–3)	4 (3–4)	4 (4–5)*	4 (3–5)*	2 (2–3)*	3 (2–3)*
	Control group (*n* = 30)	3 (2–3)	4 (3–5)	5 (4–6)	5 (4–6)	5 (4–7)	3 (3–4)

*Ai-PCIA* artificial intelligent patient-controlled intravenous analgesia

*P < 0.05 compared with control group

### RCSQ scores

As shown in Table 3, the RCSQ score of Ai-PCA group was significantly higher than that of control group on the 1st and 2nd days after operation (all *P* < 0.05) (Table 4).

**Table 3:** Comparison of RCSQ scores at different time points between the two groups of patients [M(IQR)]

Items	Ai-PCA group (*n* = 30)	Control group (*n* = 30)	*P*
1 day before surgery	65.8 (47.8–75.6)	58.6 (49.5–73.8)	0.069
Day 1 after surgery	62.9 (53.2–72.5)	52.4 (48.1–59.6)	0.024
Day 2 after surgery	68.6 (61.8–74.7)	57.8 (52.0–62.4)	0.017

*Ai-PCIA* artificial intelligent patient-controlled intravenous analgesia

**Table 4:** Comparison of analgesic pump compression, total sufentanil dosage, rescue analgesia cases, and postoperative hospital stay

Items	Ai-PCA group (*n* = 30)	Control group (*n* = 30)	*P*
Number of analgesic pump compressions	9.0 (8.0–11.2)	9.0 (6.7–12.0)	0.116
Total sufentanil dosage	7.5 (7.0–9.2)	8.0 (6.7–10.0)	0.074
Rescue analgesia cases	10 (33.3%)	7 (23.3%)	0.112
Postoperative hospital stay (days)	4.6 ± 4.0	9.2 ± 5.0	0.058

*Ai-PCIA* artificial intelligent patient-controlled intravenous analgesia

### Adverse events and outcomes

As shown in Table 4, there was no significant difference in the number of analgesic pump compressions, total sufentanil dosage and rescue analgesia cases, length of hospital stay between the two groups within 48 h after surgery (all *P* > 0.05). None of the two group patients used dezocine for rescue analgesia within 48 h after operation.

As shown in Table 5, there were no significant differences in the incidence of dizziness and nausea, vomiting, and myocardial ischemia between two groups (all *P* > 0.05).

**Table 5:** Comparison of adverse events in the two groups within 48 h after surgery

Items	Ai-PCA group (*n* = 30)	Control group (*n* = 30)	*P*
Dizziness	5 (16.7%)	7 (23.3%)	0.109
Nausea and vomiting	6 (20.0%)	11 (36.7%)	0.087
Myocardial ischemia	0 (0%)	3 (10.0%)	0.057

*Ai-PCIA* artificial intelligent patient-controlled intravenous analgesia

## Discussion

Currently, opioid-based PCIA is still the most commonly used analgesia for postoperative analgesia after laparoscopic colorectal surgery [24]. Ai-PCIA management is a dynamic management system based on information technology. Through more refined and standardized pain management, compared with traditional PCIA, Ai-PCIA can significantly improve the analgesic effect [25, 26]. The results of this study have shown that compared with traditional PCIA, Ai-PCIA can significantly decrease NRS scores at rest and during activities in the early postoperative period, and significantly improve the postoperative sleep quality.

Optimizing postoperative analgesia is the core of accelerated surgical rehabilitation, especially improving the analgesic effect during activities, which is of great significance for older patients to participate in rehabilitation programs early, restore body function as soon as possible, and reduce postoperative complications [27–29]. However, the analgesic pump seriously affects the management efficiency due to its wide dispersion and lag in feedback information [30]. The previous study [31] has shown that the wireless analgesia system can predict postoperative moderate to severe pain with 90% sensitivity and 89% specificity through the signals of “insufficient analgesia” or “poor analgesia”, indicating that the system can be used as a new method for pain assessment by taking advantage of remote monitoring to actively obtain information and improve management efficiency. The wireless analgesia system’s setting for "inadequate analgesia" provides a reference for anesthesiologists to more accurately adjust the parameters of the analgesic pump [32–34]. Studies [35, 36] have shown that increasing the background dose may have potential drug accumulation and increase the risk of adverse reactions. For older patients, this study has only adjusted a single additional dose when adjustment of analgesic pump parameters is required, without increasing the background dose. Timely and precise adjustment of parameters to provide adequate analgesia is beneficial for patients to get out of bed early and promote patient recovery.

Postoperative sleep disturbance not only causes hyperalgesia to aggravate postoperative pain, but also increases the risk of postoperative cognitive dysfunction, postoperative delirium, and depression, and seriously delays postoperative recovery [37–39]. Although postoperative sleep disturbance is associated with various perioperative factors, postoperative pain may be the most important factor [40, 41]. Previous study [42] has shown that, compared with the traditional group, the wireless analgesia management group has no statistically significant difference in sleep quality on the day of surgery and the first day after surgery, and the sleep quality on the second day after surgery is significantly improved. The results of this study have shown that the sleep quality of the Ai-PCIA group on the 1st and 2nd days after surgery is significantly higher than that of the control group, indicating that intelligent PCIA can reduce the unpleasant experience caused by noxious stimulation by improving the analgesic effect to reduce patient anxiety and reduce the stress level caused by surgery.

Although opioid use is a risk factor for postoperative nausea and vomiting, opioids remain one of the safest and most effective options for relieving moderate to severe pain in older patients [43]. Due to the changes in pharmacokinetics and pharmacodynamics caused by aging, the safe medication window of older patients is narrowed, so the use of opioids should follow the principle of titration [44–46]. However, the key to postoperative pain management is the need for repeated assessments of pain levels to continuously adjust the analgesic regimen to provide adequate analgesia without increasing adverse effects [47, 48]. The results of this study have shown that the analgesic effect of older patients using Ai-PCIA is better than that of older patients using traditional PCIA, but there is no statistical difference in the dosage of sufentanil, the number of patients with rescue analgesia, nausea, vomiting, and dizziness between the two groups. The intelligent analysis of data by the wireless analgesia system solves the difficulties in the traditional management mode, and it can provide the better pain management under dynamic monitoring.

This study has certain limitations that merit discreet considerations. First, this is a single-center study with few cases. Although no differences have observed in the control population and the study population, there are only 9 women in Ai-PCA group; studies with larger sample size are needed. Second, the most important side effect in this population is the delirium. We did not prepare to collect delirium-related data in advance and lack the related data, which needs to be further investigated in the future. Third, we did not prospectively collect those data regarding respiratory problems, skin pruritis, arrhythmias, myocardial ischemia, heart failure, etc. during the interventions; those results are important outcomes that merit for further evaluation. Finally, we have found that the postoperative hospital stay in Ai-PCA group is longer, which may be biased by the small sample size. This result should be treated with caution; it is unlikely that an Ai-PCIA using less sufentanil and more rescue analgesia is able to reduce the hospital stay this much. We have found that postoperative hospital stay in the Ai-PCIA group in some patients is much longer, which may bias the finding; more studies regarding the effects of Ai-PCIA on the length of hospital are needed. Besides, it has been reported that female patients may be a risk factor for postoperative nausea and vomiting [49], and the proportion of male patients in both groups in this study was higher, which may have an impact on the results of the study. In the future, high-quality RCTs with larger sample size in different areas are needed to further explore the effects and safety of Ai-PCIA in clinical analgesia.

## Conclusions

In summary, Ai-PCIA is beneficial to improve the analgesic effect of older patients after laparoscopic radical resection of colorectal cancer, improve sleep quality, and improve the early postoperative outcome of older patients. In the future, a large-sample high-quality RCT should be conducted to further evaluate the role of Ai-PCIA to improve the prognosis of patients.

## Data Availability

All data generated or analyzed during this study are included in this published article.
